# Are northern communities an overlooked source of microplastics and tire wear particles in the Arctic?

**DOI:** 10.7717/peerj.20237

**Published:** 2025-10-23

**Authors:** Kelly Evans, Liisa Jantunen, Julian Aherne

**Affiliations:** 1 School of Environment, Trent University, Peterborough, Ontario, Canada; 2 Air Quality Processes Research Section, Environment and Climate Change Canada, Egbert, Ontario, Canada

**Keywords:** Atmospheric microplastics, Road dust, Canadian Arctic, Fourier-transform infrared spectroscopy

## Abstract

Microplastic particles (plastic 1 µm to 5 mm in length) are a contaminant of emerging concern in Arctic environments; nonetheless, few studies have evaluated atmospheric microplastics in Arctic communities. This study investigated microplastics and tire wear particles across 16 sites in the community of Iqaluit, Nunavut (population = 7,429) using road dust as an indicator of atmospheric microplastic deposition (size detection limit >50 µm). The mean concentration of microplastics (excluding tire wear particles), ranged from 36.5 ± 68.4 µg/g (5.41 ± 4.69 n/g) in industrial sites and 73.4 ± 121 µg/g (6.21 ± 4.46 n/g) in commercial sites and non-fibrous microplastics (*i.e*., fragments, films, and foams) were dominant across the study area. Various polymers were identified using Fourier-Transform Infrared spectroscopy in Attenuated Total Reflectance down to a particle size of 100 µm. The dominant polymers being polyethylene terephthalate (15%), polyester (15%), polymethyl acrylate (15%), and polystyrene (15%). Further, based on the results of the microplastic diversity integrated index, commercial and industrial regions were composed of unique microplastic communities. The mean concentration of tire wear particles (dominated by rubber; 27%) in road dust was significantly greater than other microplastics, ranging from 83.2 ± 49.1 µg/g (49.3 ± 30.0 n/g) in industrial sites to 481 ± 514 µg/g (102 ± 132 n/g) in commercial sites. The concentration of microplastics and tire wear particles in Iqaluit was consistent with observations from metropolitan cities, suggesting Arctic communities may be a substantial local source of atmospheric microplastics and tire wear particles to surrounding Arctic ecosystems.

## Introduction

The exponential growth in plastic production since the 1950’s coupled with the mismanagement of waste has resulted in a plastic pollution crisis, evidenced by the ubiquity of microplastic particles (plastic 1 µm to 5 mm in length; MacLeod et al., 2021). Microplastics are complex contaminants generally characterized by their physical and chemical attributes, including morphology (*e.g*., fibres, foams, fragments, films, and beads), size, and polymer composition (Cole et al., 2011; Rochman et al., 2019). Primary microplastics are manufactured to be micro-sized (*e.g*., plastic microbeads as exfoliants in personal care products and pre-production pellets or nurdles; Rochman et al., 2019). Secondary microplastics are formed through chemical, physical, and biological fragmentation of plastic debris, including the shedding of synthetic fibres, especially during laundering (Rochman et al., 2019). Microplastics have various sources, for instance, the abrasion of vehicle tires on road surfaces, and the flaking and chipping of automotive paint (Diana, Chen & Rochman, 2025; Xu, Rillig & Waldman, 2022). Further, microplastics are transported to local or distant environments through several pathways, including atmospheric transport and deposition (Allen et al., 2022, 2019; Dris et al., 2016; Mutshekwa et al., 2025; Roblin et al., 2020; Welsh et al., 2022).

Microplastics are readily entrained into the atmosphere through tire wear and vehicle turbulence (Brahney et al., 2020; Özen & Mutuk, 2025), soil tilling (Koutnik et al., 2021), wave breaks (Allen et al., 2020), electric forces (Zhang et al., 2025) and wind (Rezaei et al., 2019). Airborne microplastics are further deposited into local or distant environments through wet or dry deposition processes (Bergmann et al., 2019; Evangeliou et al., 2020). Atmospheric microplastic particles have received increasing attention in urban cities during the past decade as urban environments are significant contributors to atmospheric microplastic particles (Wright et al., 2020; Dris et al., 2016; Mutshekwa et al., 2025). Nonetheless, the contribution of urbanized Arctic communities to atmospheric microplastics remains understudied, thus limiting our understanding of the potential local sources of microplastics that exist in the Arctic. Despite the recognition that microplastic particles are emerging contaminants of concern in Arctic environments, there is a paucity of studies on atmospheric microplastics in Arctic communities (AMAP, 2021; Bergmann et al., 2022).

Road dust is present in every urban environment, and it is composed of natural organic and inorganic materials from diverse sources, including atmospheric deposition (Gunawardana et al., 2012), and it has been widely assessed as a passive indicator of atmospheric microplastic and tire wear deposition (Dehghani, Moore & Akhbarizadeh, 2017; O’Brien et al., 2021; Patchaiyappan et al., 2021). Road dust is a suitable matrix to monitor for microplastics in the Arctic because it allows a greater area to be surveyed in a relatively short period with minimal field equipment, compared to active atmospheric sampling that requires fixed secure locations with electricity for deployment, which is challenging in Arctic environments.

The objective of this study was to investigate the concentration, characteristics, and deposition of microplastics and tire wear particles in an Arctic community, using road dust as an indicator of atmospheric microplastic deposition. During the summer of 2022, road dust was collected from commercial (roadsides and parking lots) and industrial (roadsides) areas in Iqaluit (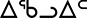), Nunavut, and assessed for microplastics and tire wear particles. Given the slower vehicle velocity in parking lots, we predicted that parking lots would have a greater concentration of microplastics and tire wear particles than roadside sites. Further, due to the unique activities that occur in commercial areas, we assumed that the diversity of microplastics in commercial and industry regions would differ. We predicted that commercial areas would have a greater diversity of microplastics than industrial areas due to increased anthropogenic presence. Herein, tire wear particles are presented separately from microplastics (*i.e*., non-rubber fibres, fragments, films, foams, and beads), despite the recognition that tire wear particles have been categorized as microplastics (Knight et al., 2020).

## Materials and Methods

### Study area and site selection

Iqaluit, Nunavut (63.7467°N, 68.5170°W), is situated in Koojesse inlet in the northwest corner of Frobisher Bay in northern Canada. Iqaluit is the largest community in Nunavut with an area of 52.5 km^2^ and a population of approximately 8,000. The community has few paved roads, no traffic lights, no stormwater drains, and <8,000 registered vehicles (dominated by trucks, all-terrain vehicles, and snowmobiles). Iqaluit is surrounded by barren land with low vegetation and a polar tundra climate that is strongly influenced by the Labrador Current. The average summer temperature during 2020–2022 was 6.1 °C (June to September) with an average wind speed of 3.6 m/s from the south-east (Government of Canada, 2023). In contrast, the average winter temperature during 2020–2022 was –18.9 °C (December to March) with an average wind speed of 4.3 m/s from the north-west (Government of Canada, 2023). The average annual precipitation in Iqaluit was ~217 mm between 2020–2022 (Government of Canada, 2023). Prior to the study period (14–15 July 2022), the highest precipitation event was on June 24^th^, 2022 (0.6 mm; Fig. S1) and the last precipitation event was on July 6^th^, 2022 (0.05 mm; Fig. S1; Government of Canada, 2023).

**Figure 1:**
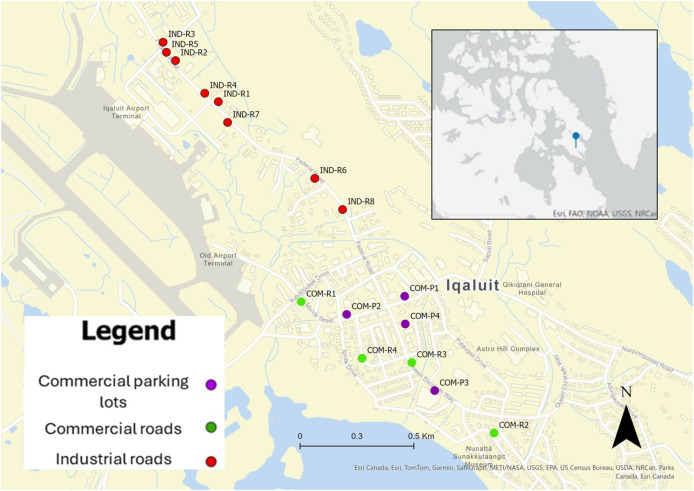
Site area. Location of study sites for commercial parking lots (COM-P; *n* = 4), commercial roadsides (COM-R; *n* = 4), and industrial roadsides (IND-R; *n* = 8), Iqaluit, Nunavut. Inlet map shows the location of Iqaluit in Canada (ArcGIS Pro, Version 3.1.1; Copyright 1999-2025 Esri).

The study sites were randomly selected from commercial and industrial areas in Iqaluit (Fig. 1; Table 1) by subdividing each region into equal area sampling units using k-means clustering of 10 m by 10 m land cover classified grids (Spatial Coverage Sampling and Random Sampling from Compact Geographical Strata; Version 0.4-2; Walvoort, Brus & Gruijter, 2023). A total of eight commercial sites were selected from parking lots (*n* = 4) and roadsides (*n* = 4), and eight industrial sites were selected from roadsides. Commercial sites were located within the community downtown core, while the industrial sites were clustered to the northwest of the community (due to limited paved surfaces in industrial regions). Commercial areas consisted of storefronts, recreational centers, and a hotel, while industrial areas consisted of construction enterprises, fuel depots, automobile shops, and storage yards. Field surveys were approved by the Nunavut Research Institute (Scientific Research License 01 011 22R-M/01 015 23R-M).

**Table 1:** Sample location and transect information.

Site ID	Date	Latitude	Longitude	Transect length	Total road dust swept	Road dust per square-meter
		dd	dd	m	g	g/m^2^
COM-R1	14-Jul-22	63.74968	−68.52949	30	45.2	3.49
COM-R2	14-Jul-22	63.74444	−68.51241	10	158	36.57
COM-R3	14-Jul-22	63.74725	−68.51968	10	22.9	5.30
COM-R4	14-Jul-22	63.74743	−68.52410	30	71.8	5.54
COM-P1	14-Jul-22	63.74987	−68.52027	10	217	50.23
COM-P2	14-Jul-22	63.74917	−68.52545	10	378	87.50
COM-P3	14-Jul-22	63.74613	−68.51767	10	137	31.71
COM-P4	15-Jul-22	63.74877	−68.52023	10	84.2	19.49
IND-R1	15-Jul-22	63.75760	−68.53675	30	8.4	0.65
IND-R2	15-Jul-22	63.75924	−68.54053	30	2.0	0.15
IND-R3	15-Jul-22	63.75997	−68.54162	30	44.4	3.43
IND-R4	15-Jul-22	63.75794	−68.53795	30	85.6	6.60
IND-R5	14-Jul-22	63.75957	−68.54134	10	18.3	4.24
IND-R6	14-Jul-22	63.75455	−68.52820	30	50.2	3.87
IND-R7	14-Jul-22	63.75678	−68.53593	30	15.5	1.20
IND-R8	14-Jul-22	63.75331	−68.52574	30	30.4	2.35

**Note:**

Site ID, sampling date (July 2022), longitude (decimal degree; dd), latitude (decimal degree; dd), transect length (meters; m), mass of total road dust collected (grams; g), and the deposition of road dust per square-meter across all sites (*n* = 16) Iqaluit, Nunavut.

### Quality assurance and quality control

Given the ubiquity of microplastics, strict quality assurance and quality control is vital to ensure that concentrations recorded reflect environmental concentrations. In the field, samples were collected against the wind and between sample sites, sampling equipment was wiped with 100% cotton cheesecloth and sprayed with compressed air to clean and to prevent contamination between sites. In the laboratory, to prevent the contamination of microplastics into environmental samples, all solutions, including reverse osmosis water, hydrogen peroxide, and sodium bromide were filtered through glass-fibre filters (Fisherbrand™ Glass Filter Circle, 1.6 µm pore diameter, G6, filter diameter 4.25 cm). A 100% cotton laboratory coat was worn, and hands/gloves were rinsed with filtered reverse osmosis (FRO) water throughout the extraction process to prevent contamination of the samples. Metal and glass equipment were used where possible to prevent potential contamination, and all equipment was triple-rinsed with FRO water before and in between the handling of environmental samples to remove adhered microplastics. Laboratory blanks (*n* = 2) were performed in sequence with environmental samples and consisted of running solutions through the same laboratory processes as environmental samples (*i.e*., digestion and density separation) to quantify potential contamination of environmental samples during the extraction process. No microplastics were detected in laboratory blanks.

Field blanks (*n* = 6) were performed at a random subset of the commercial and industrial study sites and mimicked the field sampling procedures by sweeping microplastic-free road dust (250 g pre-baked at 500 °C for 12 h) off a cork board into the sampling containers. Field blanks were used to capture any potential microplastic or tire wear particle contamination that occurred during the sweeping process (*e.g*., atmospheric deposition, insufficient cleaning of field equipment). Further, field blanks (1 g) were also spiked with a known concentration of polyethylene (PE) beads (size ranges 75–90 and 212–250 µm) to assess recovery rates and visual limits of detection; blanks were processed in sequence with environmental samples. The recovery for spiked PE microbeads from the field blanks (*n* = 4) ranged from 70–100% (average = 83%) for sizes 75–90 µm and 90–100% (average = 98%) for sizes 212–250 µm, which is consistent with reported recovery rates (Dimante-Deimantovica et al., 2024). Samples were not corrected based on microbead recovery. The concentrations of microplastics (0.17 n/g) in the field blanks were low and given that all observed concentrations were greater than the limit of detection (LOD; 1.4 n/g), microplastic observations were not blank-corrected. No tire wear particles were detected in field blank samples. The LOD was estimated as the mean microplastic count for field blanks plus three times their standard deviation (Bertrim & Aherne, 2023). Open-air laboratory blanks (*n* = 2) consisted of an exposed glass-fibre filter in a Petri dish beside the microscope to capture microplastics from the indoor atmosphere. No microplastics were detected in open-air blanks.

### Sample collection

On 14–15 July 2022, road dust was collected using a natural-fibre straw brush and a metal dustpan (O’Brien et al., 2021). Briefly, at each site the pavement was swept along a 10−30 m transect (width = 43.2 cm, length of transect depended on quantity of road dust available). For roadside sites, the start of the transect was 43.2 cm from the shoulder of the road. For parking lot sites, samples were collected from parking spaces. Road dust was collected against the direction of the wind. All contents in front of the metal pan (width = 43.2 cm) were collected using a slow sweeping motion to minimize the resuspension of microplastic particles. Road dust contents in the dustpan were sieved at 2 mm to remove large rocks and non-plastic debris, and the <2 mm fraction was retained in an aluminum dish (pre-baked at 400 °C for 4 h), which was subsequently covered and wrapped with aluminum foil and stored in a brown paper bag until analysis.

### Microplastic extraction

Road dust samples were first oven-dried at 45 °C for 48 h, homogenized by gently stirring (~1 min), and 1 g of road dust from each site was processed in 0.5 g aliquots to optimize the analytical procedure (*i.e*., a total of 8 g each for commercial and industrial areas). Then, organic matter was removed using wet oxidation; in a 250 mL Erlenmeyer flask, 40 mL of filtered 30% hydrogen peroxide was added to each road dust sample and digested at 45 °C for 24 h (Hurley et al., 2018). Following digestion, samples were wet sieved (20 µm), and the residue was retained in a 100 mL tall glass beaker by rinsing the sieve with FRO water. The filtrate was retained from a subset of samples (*n* = 8) to determine if there was particle loss from the sieving process; no microplastics or tire wear particles were detected in the filtrate. Next, microplastics in the residue were subsequently extracted using one water density separation. The water density separation was accomplished by filling each tall beaker with FRO water (~75 mL) and stirring samples on a stir-plate for ~1 min. After the samples were stirred, the walls of the beaker were rinsed with FRO to wash adhered particles off the beaker wall, and the beaker was filled to the 100 mL line with FRO. The beakers were left on the laboratory bench at ambient temperature for 24 h to allow particulates to settle. Following the settling time, samples were filtered by washing the beaker walls with FRO water as the supernatant was decanted onto a glass-fibre filter. This process reduced the surface tension pull of particles and optimized the extraction process. Filters were stored in clear polystyrene petri dishes until visual analysis. The density separation process was repeated twice using filtered sodium bromide (NaBr; density =1.5–1.6 g/cm^3^) in replacement of FRO water.

### Microplastic and tire wear particle identification

Filters were visually analyzed for microplastics and tire wear particles using a stereomicroscope (AmScope version x64). The entire filter surface was analyzed for microplastics, which were identified using the following set of criteria: (1) particles were unnaturally coloured relative to the sample, and appeared homogeneous in material and texture with no visible cellular structures, (2) fibres were a consistent width throughout, (3) particles remained intact when compressed or poked with dissection instruments, (4) particles had a shiny or glossy appearance, and (5) fibres shared no similarities to natural fibres with limited fraying (Kosuth, Mason & Wattenberg, 2018). Given the abundance of tire wear particles, a quarter of the filter surface was analyzed and scaled up. Tire wear particles were identified as being (1) black, (2) elongated/cylindrical in shape, (3) with a rough surface texture, and (4) spongy when poked with a dissection instrument but remained intact (Leads & Weinstein, 2019).

All suspected microplastic or tire wear particles that met at least two visual identification criteria were photographed (AmScope MU100 camera, 3,584 × 2,748 pixels). Suspected microplastics were touched with a heat source (400 °C; Beckingham et al., 2023) while suspected tire wear particles were not touched with a heat source as they do not melt at high temperatures. The length and width of all microplastics that melted were measured using AmScope (Version 4.11.22004.20230115); 95% of the observed tire wear particles were measured. The average length and diameter per particle shape from each site were used for particles with no measurements. Further, a 540 nm blue light (NightSea Stereo Microscope Fluorescence Adapter) was used to capture fluorescent particles difficult to see under a standard brightfield light. In general, visual identification is limited to particles >50 µm, but where possible, particles down to ~20 µm were also identified, albeit a small proportion (~5%). It is important to note that one individual completed all visual analysis and detection favoured microplastics >50 µm due to visual limitations. Fourier-Transform Infrared spectroscopy (FTIR; LUMOS II, Brucker) was performed on a subset of suspected microplastic and tire wear particles using attenuated total reflection (ATR) to determine polymer type. The particles analyzed by ATR-FTIR were not melted and they were selected randomly. A total of 30% microplastics and 22% tire wear particles were picked with clean tweezers and transferred to a slide with double-sided tape (Thornton Hampton et al., 2022). The diameter of the ATR crystal (Germanium; diameter of tip = 100 µm) generally limited polymer analysis to larger particles (>100 µm); fibrous microplastics were particularly challenging due to their narrow diameter. For all particles, a scan time of 16 s was used under low pressure. Subsequently, tire wear particle spectra were corrected for ‘Black Rubber’ in OPUS (Version 8.7). Despite correcting the tire wear particle spectrum, it is important to note there are limitations to using ATR-FTIR for spectral identification of tire wear particles as their dark nature only reflects a small amount of light (Kang et al., 2022). All spectra were uploaded to OpenSpecy to identify polymer type (Cowger et al., 2021). The plastic polymer with the highest Pearson’s Correlation was chosen, with a minimum correlation value of >0.5 due to the challenges of spectroscopic analysis of aged microplastics and tire wear particles.

### Data analysis

Field sites were grouped into industrial roadsides (IND-R), and commercial sites (COM-P+R), with the latter comprising commercial parking lots (COM-P) and commercial roadsides (COM-R). The concentration (n/g) of microplastics and tire wear particles per gram dry weight (dw) of road dust was estimated for each site by summing counts from each duplicate and dividing by the sum of road dust mass from each site (~1 g). The concentration of microplastics and tire wear particles were polymer corrected by multiplying the estimated concentration by the proportion of particles identified as plastic by ATR-FTIR. The proportion of polymers confirmed as plastic by ATR-FTIR was calculated by dividing the number of microplastics or tire wear particles confirmed as plastic by the total number particles analyzed by ATR-FTIR and converted to percentage. In this study, the proportion of particles confirmed as plastic was 75% for microplastics and 87% for tire wear particles (see Table S4). Therefore, concentrations of microplastics and tire wear particles were multiplied by 0.75 and 0.87, respectively. It is important to note that the estimated concentrations of microplastics and tire wear particles represent particles that generally ranged from 20–2,000 µm, however, visually identifying microplastics <50 µm was challenging due to their small size.

Mass concentrations (µg/g) were estimated for both microplastics and tire wear particles by using the volume of each particle (see Table S1 for equations used to calculate the volume of microplastics by shape) and the average polymer density (Simon, van Alst & Vollertsen, 2018). Reporting count and mass concentration develops a concrete understanding on the quantity of plastic in diverse environments. This is especially important because microplastics and tire wear particles exist in unique shapes and sizes, and this influences their mass abundance. The volume of microplastics and tire wear particles was calculated for each shape at all sites (see Table S1). Despite the irregular and diverse shapes of microplastic, all microplastics and tire wear particles were simplified to three-dimensional objects to estimate mass concentrations. Mass was calculated by summing the volume by particle shape and multiplying by the average density for shape-specific polymers (see Table S2).

Further, deposition was calculated by dividing the total mass (g) of road dust collected at each site by the sampling area (area (m^2^) = width of dustpan × transect length; Table 1) and multiplying that value by the concentration of microplastics or tire wear particles (n/g or µg/g). We assumed concentration and deposition represented an accumulation of atmospheric microplastics and terrestrial particles directly emitted from their sources (such as tire wear particles). Hereafter, concentration and deposition (± standard deviation) are reported as per gram dry weight of road dust and per square metre, respectively. The data was not significantly different from normal (Shapiro-Wilk test); therefore, a one-way analysis of variance (ANOVA) was used to test for a statistical difference between the concentration and deposition of microplastics and tire wear particles in commercial and industrial regions. A confidence interval of 95% was used, followed by a Tukey’s test if there was statistical significance. An ANOVA was also used to test for statistical differences between microplastic and tire wear particle characteristics including shape and length across commercial and industrial regions. Further, to estimate the microplastic diversity across groups, a microplastic diversity integrated index (MDII) was used (Li et al., 2021). Briefly, a Simpson’s 1-D index was used to estimate the diversity of microplastic shape, colour, and size bins in road dust samples from commercial roadsides and parking lots, and industrial roadsides. The microplastic community composition was calculated for each group by multiplying the Simpson’s 1-D index results for shape, colour, and size together and taking the cubic root of the value obtained (Li et al., 2021). All figures and statistical analyses were carried out in the software Past (Version 4.12; Hammer, Harper & Ryan, 2001).

## Results

### Microplastics in Arctic road dust

#### Concentration and deposition

**Figure 2:**
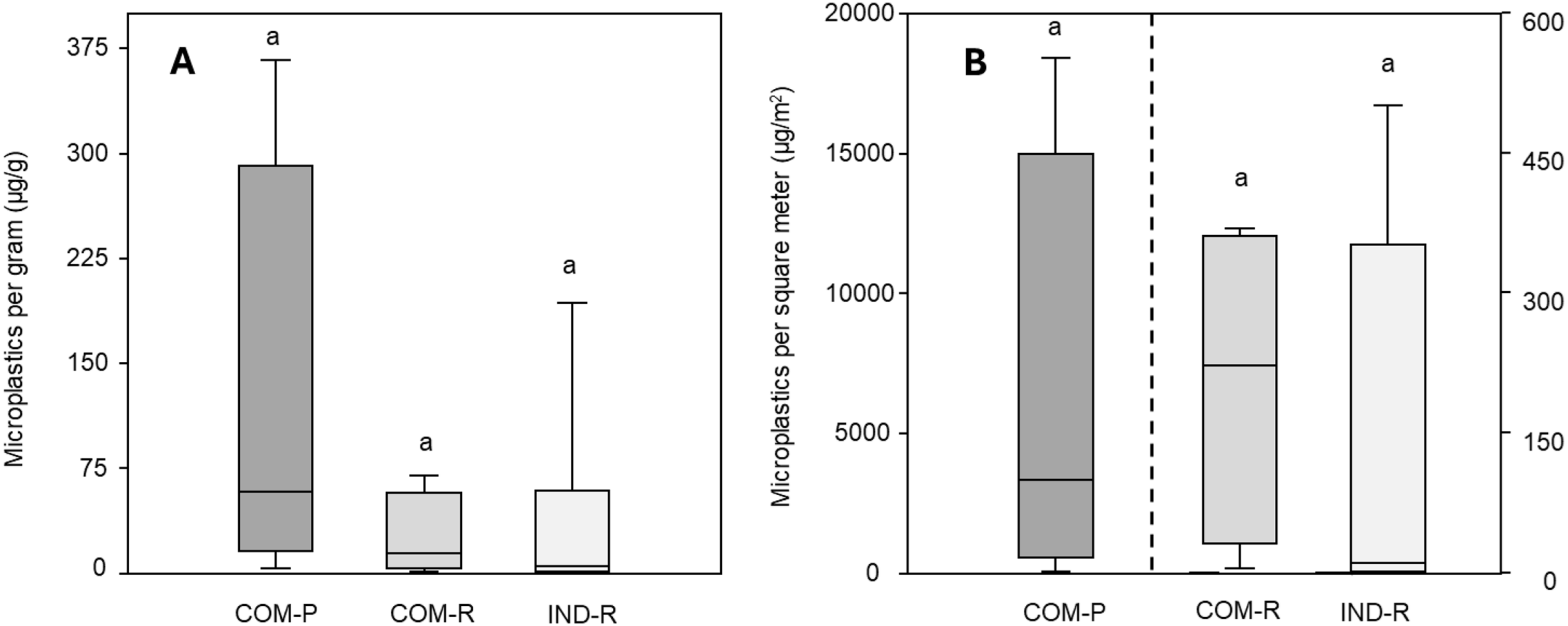
Mass concentration and deposition of microplastics in Arctic road dust. Box plots present the distribution of the (A) concentration (µg/g) and (B) deposition (µg/m^2^) of microplastics in commercial parking lots (COM-P; *n* = 4), commercial roadsides (COM-R; *n* = 4), and industrial roadsides (IND-R; *n* = 8) across Iqaluit, Nunavut. Lowercase letters indicate statistical significance (*p* < 0.05). The dotted line separates the two axes to improve readability in plot B. The box represents the 25th and 75th percentile, the horizontal line represents the median and the whiskers represent the interquartile range.

In total, 102 suspected microplastics (see Table S3 for character information on all suspected particles and Table S4 polymer matches across areas) were identified at 15 of the 16 study sites. The mean concentration of microplastic particles (excluding tire wear particles) was 55.0 ± 97.3 µg/g (5.80 ± 4.44 n/g) across the study area. The concentration was 36.5 ± 68.4 µg/g (5.41 ± 4.69 n/g) in industrial sites and 73.4 ± 121 µg/g (6.21 ± 4.46 n/g) in commercial sites, ranging from 25.0 ± 30.7 µg/g (4.03 ± 4.33 n/g) in commercial roadsides to 122 ± 165 µg/g (8.38 ± 3.89 n/g) in commercial parking lots (See Table S5 for concentrations across sites). There was no statistical difference in count or mass concentrations across groups *(p* > 0.05; Fig. 2).

Microplastic deposition followed the same pattern; the mean across all sites was 1,688 ± 4,622 µg/m^2^ (130 ± 248 n/m^2^) with a mean deposition of 3,250 ± 6,338 µg/m^2^ (17.3 ± 20.2 n/m^2^) in industrial sites and 3,250 ± 6,338 µg/m^2^ (242 ± 319 n/m^2^) in commercial sites, ranging from 204 ± 176 µg/m^2^ (36.2 ± 31.6 n/m^2^) in commercial roadsides to 4,152 ± 8,304 µg/m^2^ (448 ± 352 n/m^2^) in commercial parking lots (See Table S5 for deposition across sites). There was a statistical difference between groups, the count deposition of microplastics were significantly greater in commercial parking lots than in both commercial and industrial roadsides (*p* < 0.05).

#### Characteristics (Morphology, colour, size, and polymer composition)

Based on visual analysis, blue fragments were the dominant microplastic particle identified across commercial parking lots (25%) and industrial roadsides (56%) and blue fibres were dominant in commercial roadsides (33%; see Table S6). Overall, a greater proportion of non-fibrous microplastics (*i.e*., fragments, films, foams) were present compared to microplastic fibres across the study area, however, this difference was not significant *(p* > 0.05). The mean (range) length of fibrous microplastics were not statistically different (*p* > 0.05) across the study area, ranging from 792 ± 346 µm (339–1,264 µm) in commercial parking lots, to 1,169 ± 823 µm (201–2,097 µm) in industrial roadsides, and 1,157 ± 560 µm (547–2,040 µm) in commercial roadsides. The mean (range) length of non-fibrous microplastics ranged from 121 ± 82.5 µm (19.2–316 µm) industrial roadsides, to 182 ± 74.2 µm (67.1–294 µm) in commercial roadsides, and 272 ± 307 µm (62.1–1,223 µm) in commercial parking lots. Overall, commercial areas had a greater abundance of microplastics between 101–500 µm in length, while industrial roadsides had a greater abundance of microplastics <100 µm in length (see Table S6).

Various common polymers were identified across the subset of particles analysed by ATR-FTIR in commercial parking lots, commercial roadsides, and industrial roadsides. Across the study area polyester (40%) was the dominant polymer for microplastic fibres, polymethyl acrylate (50%) was the dominant polymer for fragments, polystyrene (100%) was dominant for films, and polyvinyl butyral, polyurethane, and polyvinyl chloride were equally dominant for foams (Table 2). The results from the Microplastic Diversity Integrated Index suggest that the microplastic diversity in commercial parking lots (0.68) and commercial roadsides (0.67) were nearly identical and they both differed (greater diversity) from industrial roadsides (0.51; Table 3).

**Table 2:** Identified polymers for microplastic morphologies.

Morphology	Polymer type	Proportion (%)
Fibre	Polyester	40
	Polymethyl acrylate	20
	Polyethylene	20
	Polyethylene terephthalate	20
Fragment	Polymethyl acrylate	50
	Polyethylene terephthalate	25
	Polystyrene	25
Film	Polystyrene	100
Foam	Poly(vinyl butyral)	33
	Polyurethane	33
	Polyvinyl chloride	33
Tire wear particle	Rubber	27
	Polypropylene	20
	Polyethylene	18
	Polystyrene	16
	Polyvinyl chloride	12
	Nitrile	2
	Poly(vinyl butyral)	1
	Polyester	1
	Polymethyl acrylate	1
	Polyethylene terephthalate	1
	Polyacrylate nitrile	<1
	Low-density polyethylene	<1
	Polyurethane	<1

**Note:**

Proportion of identified polymers for microplastic fibres, films, foams, and tire wear particles in road dust across commercial parking lots (*n* = 4), commercial roadsides (*n* = 4), and industrial roadsides (*n* = 8) in Iqaluit, Nunavut.

**Table 3:** Microplastic diversity integrated index.

Diversity	COM-P	COM-R	IND-R	COM-P+R
Shape	0.72	0.66	0.47	0.70
Colour	0.66	0.66	0.48	0.62
Size	0.66	0.69	0.60	0.66
MDII	0.68	0.67	0.51	0.10

**Note:**

Results from the Simpson’s diversity index for microplastic shape, colour, and size in road dust from commercial parking lots (COM-P; *n* = 4), commercial roadsides (COM-R; *n* = 4), and industrial roadsides (IND-R; *n* = 8) located in Iqaluit, Nunavut.

### Tire wear particles in Arctic road dust

#### Concentration and deposition

**Figure 3:**
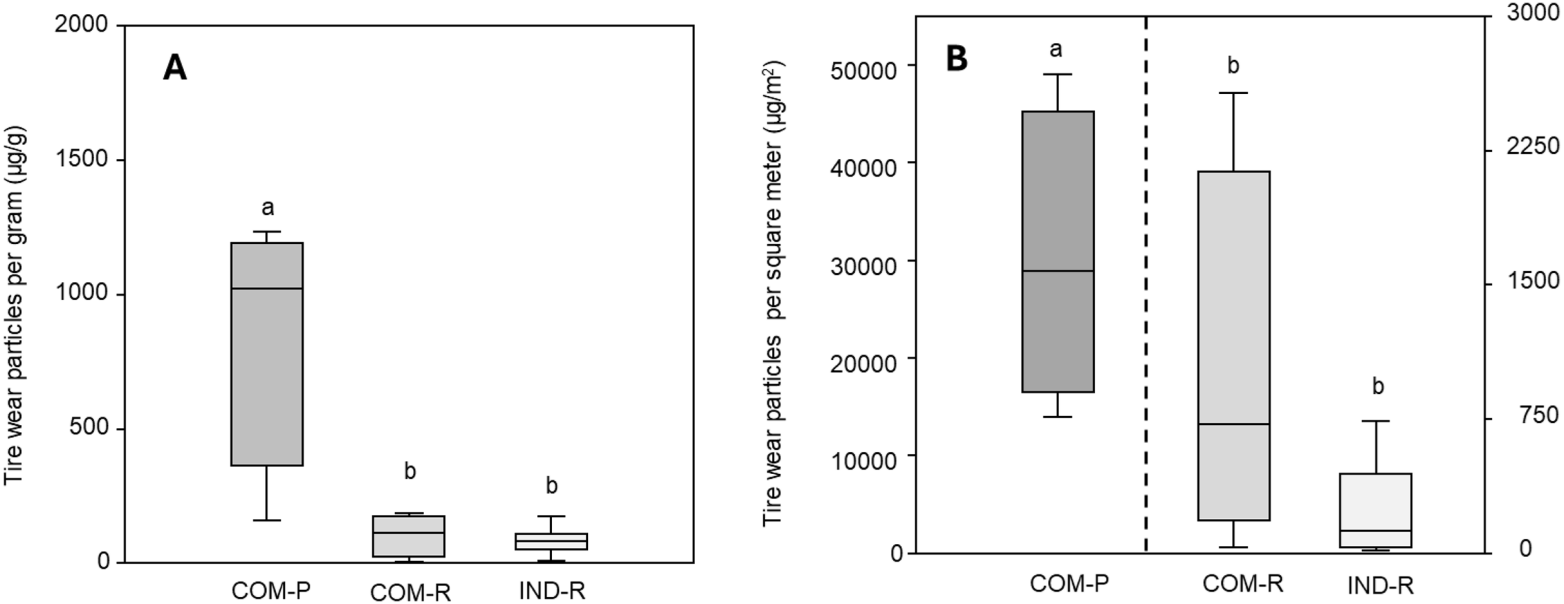
Concentration and deposition of tire wear particles. Box plots illustrating the (A) concentration of tire wear particles (µg) per gram dry weight road dust and (B) deposition of tire wear particle (µg/m^2^) from commercial parking lots (COM-P; *n* = 4), commercial roadsides (COM-R; *n* = 4), and industrial roadsides (IND-R; *n* = 8) in Iqaluit, Nunavut. The dotted line separates the two axes to improve readability. The Lowercase letters indicate statistical significance (ANOVA, *p* < 0.05). The box represents the 25th and 75th percentile, the horizontal line represents the median and the whiskers represent the interquartile range.

Tire wear particles were pervasive across the study area, and 1,317 tire wear particles were quantified across all sites (Table S5). The mean concentration of tire wear particles was 282 ± 408 µg/g (75.8 ± 101 n/g) across the study area, with a mean concentration of 83.2 ± 49.1 µg/g (49.3 ± 30.0 n/g) in industrial sites and 481 ± 514 µg/g (102 ± 139 n/g) in commercial sites, from 103 ± 80.5 µg/g (22.2 ± 6.98 n/g) in commercial roadsides, to 859 ± 478 µg/g (183 ± 166 n/g) in commercial parking lots (see Table S5 for concentration across sites). There was a statistical difference in the mass concentration of tire wear particles across groups, commercial parking lots had a greater concentration than both commercial and industrial roadsides (*p* < 0.05; Fig. 3).

The deposition of tire wear particles followed the same pattern, with a mean deposition of 7,926 ± 14,900 (2,301 ± 5,383 n/g) across the study area; ranging from 227 ± 263 µg/m^2^ (171 ± 181 n/m^2^) in industrial sites and 11,079 ± 15,644 µg/m^2^ (5,780 ± 9,340 n/m^2^) in commercial sites, ranging from 1,013 ± 1,091 µg/m^2^ (278 ± 353 n/m^2^) in commercial roadsides, to 30,235 ± 14,948 µg/m^2^ (8,584 ± 8,629 n/m^2^) in commercial parking lots (see Table S5 for deposition across sites). There was a statistical difference between groups, deposition of tire wear particles was significantly greater in commercial parking lots than commercial and industrial roadsides (*p* < 0.05; Fig. 3).

#### Characteristics (Size and polymer composition)

The mean length of tire wear particles was 151 ± 119 µm across the study area. The mean (range) length of tire wear particles was not statistically different between groups and ranged from 1,346 ± 117 µm (39.0–1,859 µm) in commercial roadsides to 129 ± 98.9 µm (31.5–955 µm) in industrial roadsides, and 164 ± 128 µm (27.0–1,859 µm) in commercial parking lots. Further, a total of 13 plastic polymers were identified for tire wear particles across sites, rubber (27%) was dominant, followed by polypropylene (18%; Table 2); however, this may reflect the challenges of spectroscopic analysis of tire wear particles.

## Discussion

The commercial sites (*i.e*., parking lots and roadsides) shared a similar microplastic diversity (*i.e*., morphology, colour, and size), and both differed from industrial roadsides (Table 3), suggesting there is a greater quantity of microplastic sources in commercial areas compared to industrial areas. Overall, the concentration of microplastics (54.97 ± 97.28 µg/g; 5.80 ± 4.44 n/g) and tire wear particles (282 ± 408 µg/g; 75.8 ± 101 n/g) in road dust from Iqaluit, Nunavut, was comparable with that of metropolitan cities, and commercial parking lots had a notably greater concentration of microplastics and tire wear particles than roadside sites. The concentration and deposition of tire wear particles were significantly greater in parking lots than on roadsides, indicating parking lots may serve as a temporary reservoir for tire wear particles. There was also a greater quantity of dust per meter-square in parking lots, indicating parking lots have a greater retention of dust than roadsides. The greater accumulation likely reflects the slower vehicle velocity in parking lots, which ultimately reduces the resuspension of particles into the atmosphere.

Given the population density of Iqaluit, microplastic concentrations in road dust were expected to be lower than in metropolitan cities as microplastic concentrations are positively correlated with population density (Yukioka et al., 2020). However, the count concentration of microplastics in road dust from Iqaluit (3.91 ± 3.02 n/g) was similar to Chennai, India (2.28 ± 0.89 n/g; population = 6.7 million; Patchaiyappan et al., 2021), Kathmandu, Nepal (3.90 n/g; population = 856,000; Yukioka et al., 2020), Da Nang, Vietnam (4.10 n/g; population = 1.1 million; Yukioka et al., 2020), and Kasatsu, Japan (2.50 n/g; population = 139,000; Yukioka et al., 2020). There are no studies that investigate microplastics and tire wear particles across north America or northern Europe, which limits comparisons to a few metropolitan areas in Asia and Australia. Further, the combined mass concentration of microplastics and tire wear particles were within the range of road dust in Brisbane, Australia (500–6,000 µg/g; population = 1.2 million; Table S5; O’Brien et al., 2021). The deposition of microplastics across Iqaluit was comparable to Kusatsu, Japan (2.00 ± 1.60 n/m^2^; population = 139,000), Kathmandu, Nepal (12.5 ± 10.1 n/m^2^; population = 856,000), and Da Nang, Vietnam (19.7 ± 13.7 n/m^2^; population = 1.1 million; Table S5; Yukioka et al., 2020). Overall, the similarities and differences in microplastic concentrations in Iqaluit from road dust compared with more densely populated cities is concerning. The concentrations of microplastics were expected to be lower in the Arctic compared to metropolitan areas, as atmospheric microplastic deposition in snow had significantly lower quantities of microplastics detected in Arctic compared to a European metropolitan city (Bergmann et al., 2019). Nonetheless, the observations in a remote northern community are surprisingly within the same order of magnitude as studies conducted within metropolitan areas. However, it may reflect differences in sampling methodologies; for example, Yukioka et al. (2020) used a vacuum cleaner to collect road dust, and samples were sieved at 75 µm, which may have resulted in the loss of fibrous microplastics, which have a narrow diameter (10–20 µm).

Dominant microplastic shape also varied across studies; a greater proportion of fibrous microplastics was reported in road dust in Bushehr City, Iran (76%; population = 220,000) using a plastic brush and pan, which was similar to commercial roadside findings in the current study (Abbasi et al., 2017). In contrast, our findings are more similar to a study in Ma’anshan City, China (population = 2.1 million), which identified fragments (50%) as the dominant microplastic shape in road dust collected using a wooden brush and steel shovel (Wang et al., 2022). A higher proportion of fragments (68–81%) was also observed in road dust sampled from Kusatsu, Japan, Da Nang, Vietnam, and Kathmandu, Nepal (Yukioka et al., 2020); however, it is likely that the field methods used by Yukioka et al. (2020) favoured the collection of non-fibrous microplastics.

Comparing the concentration and deposition of tire wear particles in road dust to other studies is challenging as tire wear particles are generally grouped within the microplastic shape category of “fragments” or excluded from studies altogether due to the challenges of visual and spectroscopic analysis. Dehghani, Moore & Akhbarizadeh (2017) investigated tire wear in road dust from Tehran, Iran (population = 7.7 million), and suggested a concentration of 16.6 n/g road dust. In the current study, the average concentration of tire wear across the study area was 303 n/g. The higher concentration of tire wear particles in Iqaluit (282 ± 408 µg/g; 75.8 ± 101 n/g) may be a result of the sampling and analytical techniques, as we had a high recovery of microbeads of all sizes in the spiked blanks; to the best of our knowledge, recovery limits were not reported in the previously mentioned studies, making comparisons challenging. However, the greater concentration in Iqaluit may be a result of the vehicle tires used in the community; for instance, the majority of vehicles in the north use winter tires that are composed of a soft rubber material and may shed more particles than all-year or summer tires (Wilkinson et al., 2023). In contrast, concentrations of tire wear particles in Goyang city, South Korea (~1,210 n/g) were greater than in the current study (Kang et al., 2022). Further, a study that investigated tire wear particles accumulated in roadside snowbanks in Norway identified an average concentration of 10,600 ± 2,200 mg/m^2^ (Rødland et al., 2022). The higher concentration of tire wear particles in snowbanks than in the current study is likely a result of the greater accumulation period throughout the winter (several months), in contrast to road dust which reflects accumulation following the most recent washout event (unknown period in the current study).

Understanding the concentration and characteristics of microplastics and tire wear particles in road dust is crucial as road dust facilitates the transport of microplastics and tire wear particles to the surrounding and distant environments. For instance, microplastics and tire wear particles become re-entrained into the atmosphere by wind or vehicle traffic and are subsequently deposited back to the ground in local or distant environments (Evangeliou et al., 2020). Microplastics in suspended dust may also be inhaled and have been detected in lung tissues. However, the associated risks are not yet well established, as most studies focus on microplastic exposure through ingestion (Abbasi et al., 2019; Baensch-Baltruschat et al., 2020). Preliminary research suggests that exposure to microplastics through inhalation may induce respiratory disease (Abbasi et al., 2019). Road dust also facilitates the transport of microplastics to aquatic systems. For example, through stormwater drainage following precipitation and washout events (Monira et al., 2021). Microplastics and tire wear particles have been observed in Arctic sediments close to communities. suggesting northern communities are a source of microplastics and tire wear particle to the surrounding water and sediment (Huntington et al., 2020; Adams et al., 2021). Microplastics and tire wear particles that enter aquatic environments have adverse effects, such as infiltrating the aquatic food chain and causing physical harm to aquatic species when ingested (Lim, 2021). Microplastics and tire wear particles are also associated with chemical additives (*e.g*., polycyclic aromatic hydrocarbons, petroleum hydrocarbons, phthalates, and dyes) that leach into surrounding environments (Andjelković et al., 2021; Gitlitz, Gartner & Vallette, 2024; Teuten et al., 2009). Such additives also have adverse effects on aquatic species, for instance, 6PPD (N-(1,3-dimethylbutyl)-N’-phenyl-p-phenylenediamine) improves the durability of tires but its oxidation product 6PPD-quinone is highly toxic to aquatic organisms (Tian et al., 2022).

We acknowledge there are limitations to our study that may influence the estimated concentration and deposition of microplastics and tire wear particles in road dust. First, our study reports microplastics and tire wear particles in road dust over an unknown accumulation period, future studies could consider deploying a pre-cleaned container to estimate deposition at each site. Second, the detection limit for microplastics in this study was between >20–2,000 µm, which resulted in the potential exclusion of microplastics between 2–5 mm in length making cross comparisons challenging, however, concentrations were still in the same magnitude. Third, the recovery of spiked microplastics may not reflect the recovery of environmental fibres, films, foams, fragments, and tire wear particles, therefore, concentrations were not corrected based on these recoveries. Finally, the mass concentrations are an estimate based on two measurements (longest length and width), given that microplastics and tire wear particles are intricate, three-dimensional particles with complex chemical structures, this may skew the estimated mass of microplastics and tire wear particles. Due to the limitations in the study, the concentration and deposition reported in this study are presented as estimates of environmental levels.

## Conclusion

In this study, we shed light on the presence of microplastics and tire wear particles in Arctic Road dust. The mean concentration of microplastics was 55.0 ± 97.3 µg/g (5.80 ± 4.44 n/g) and tire wear particles was 282 ± 408 µg/g (75.8 ± 101 n/g) in road dust across Iqaluit, Nunavut. The high magnitude of microplastics and tire wear particles in commercial parking lots compared to roadsides suggests that parking lots are a temporary reservoir (accumulation zone) for microplastics and tire wear particles. Further, the study suggests the concentration of microplastics in Arctic road dust is comparable to observed concentrations in metropolitan areas, underscoring the importance of understanding the contribution of local Arctic communities to environmental and atmospheric microplastics and tire wear particles, and their role in the transport of microplastics and tire wear particles to the wider environment. The increased attention to microplastics and tire wear particles as transboundary contaminants requires coordinated efforts for efficient monitoring across the Arctic. Further, road dust may serve as a beneficial medium to monitor atmospheric microplastic deposition in the Arctic due to the ease of sample collection and analysis.

## Supplemental Information

10.7717/peerj.20237/supp-1 Supplemental Information 1 Wind speed and precipitation. Wind speed (km/hr, blue line) and precipitation (mm, black bars) for A) June 2022 and B) July 2022 prior to sampling period in Iqaluit, Nunavut. Data obtained from Iqaluit Climate Air Monitoring Station.

10.7717/peerj.20237/supp-2 Supplemental Information 2 Equations to estimate microplastic volume. Equations used to estimate microplastic volume for fibres, films, fragments, foams, and tire wear particles, modified from Simon et al. (2018).

10.7717/peerj.20237/supp-3 Supplemental Information 3 Polymer densities. Density (gram per cubic meter; g/cm 3 ) for plastic polymers identified by Fourier-transform Infrared spectroscopy for each microplastic shape (*i.e*., fibres, fragments, films, foams, and tire wear particles). Average density was used in to calculate mass concentrations.

10.7717/peerj.20237/supp-4 Supplemental Information 4 Microplastic and tire wear particles across the study area. Each row contains characteristic data for microplastics and tire wear particles across commercial parking lots (COM-P), commercial roadsides (COM-R) and industrial roadsides (IND-R) in road dust from Iqaluit, Nunavut.

10.7717/peerj.20237/supp-5 Supplemental Information 5 Polymer matches for a subset of microplastics and tire wear particles across the study area. Each row contains polymer matches with the respective high index quality (HIQ) for microplastics and tire wear particles across commercial parking lots (COM-P), commercial roadsides (COM-R), and industrial roadsides (IND-R) in Iqaluit, Nunavut.

10.7717/peerj.20237/supp-6 Supplemental Information 6 Count and mass concentration and deposition of microplastics and tire wear particles in road dust. Each row contains the average count and mass concentration or deposition of microplastics by shape and tire wear particles for each site across Iqaluit, Nunavut.

10.7717/peerj.20237/supp-7 Supplemental Information 7 Colour and size bin counts for microplastics across the study area. Counts for the colours and size bin of microplastics identified across commercial areas (COM-P+R), commercial parking lots (COM-P), commercial roadsides (COM-R), and industrial roadsides (IND-R).

## Additional Information and Declarations

### Field Study Permissions

The following information was supplied relating to field study approvals (*i.e*., approving body and any reference numbers):

Field surveys were approved by the Nunavut Research Institute (Scientific Research License 01 011 22R-M/01 015 23R-M).

### Data Availability

The following information was supplied regarding data availability:

The raw data (microplastic particle counts) are available in the Supplemental Files.

## References

[ref-1] Abbasi S, Keshavarzi B, Moore F, Delshab H, Soltani N, Sorooshian A (2017). Investigation of microrubbers, microplastics and heavy metals in street dust: a study in Bushehr city, Iran. Environmental Earth Sciences.

[ref-50] Abbasi S, Keshavarzi B, Moore F, Turner A, Kelly FJ, Dominguez AO, Jaafarzadeh N (2019). Distribution and potential health impacts of microplastics and microrubbers in air and street dusts from Asaluyeh County, Iran. Environmental Pollution.

[ref-38] Adams JK, Dean BY, Athey SN, Jantunen LM, Bernstein S, Stern G, Diamond ML, Finkelstein SA (2021). Anthropogenic particles (including microfibers and microplastics) in marine sediments of the Canadian Arctic. Science of the Total Environment.

[ref-2] Allen D, Allen S, Abbasi S, Baker A, Bergmann M, Brahney J, Butler T, Duce RA, Eckhardt S, Evangeliou N, Jickells T, Kanakidou M, Kershaw P, Laj P, Levermore J, Li D, Liss P, Liu K, Mahowald N, Masque P, Materić D, Mayes AG, McGinnity P, Osvath I, Prather KA, Prospero JM, Revell LE, Sander SG, Shim WJ, Slade J, Stein A, Tarasova O, Wright S (2022). Microplastics and nanoplastics in the marine-atmosphere environment. Nature Reviews Earth & Environment.

[ref-3] Allen S, Allen D, Phoenix VR, Le Roux G, Durántez Jiménez P, Simonneau A, Binet S, Galop D (2019). Atmospheric transport and deposition of microplastics in a remote mountain catchment. Nature Geoscience.

[ref-39] Allen S, Allen D, Moss K, Le Roux G, Phoenix VR, Sonke JE (2020). Examination of the ocean as a source for atmospheric microplastics. PLOS ONE.

[ref-40] AMAP (2021). AMAP litter and microplastics monitoring guidelines.

[ref-4] Andjelković T, Bogdanović D, Kostić I, Kocić G, Nikolić G, Pavlović R (2021). Phthalates leaching from plastic food and pharmaceutical contact materials by FTIR and GC-MS. Environmental Science and Pollution Research.

[ref-5] Baensch-Baltruschat B, Kocher B, Stock F, Reifferscheid G (2020). Tyre and road wear particles (TRWP)—a review of generation, properties, emissions, human health risk, ecotoxicity, and fate in the environment. The Science of the Total Environment.

[ref-51] Beckingham B, Apintiloaiei A, Moore C, Brandes J (2023). Hot or not: systematic review and laboratory evaluation of the hot needle test for microplastic identification. Microplastics and Nanoplastics.

[ref-52] Bergmann M, Collard F, Fabres J, Gabrielsen GW, Provencher JF, Rochman CM, van Sebille E, Tekman MB (2022). Plastic pollution in the Arctic. Nature Reviews Earth & Environment.

[ref-6] Bergmann M, Mützel S, Primpke S, Tekman MB, Trachsel J, Gerdts G (2019). White and wonderful? Microplastics prevail in snow from the Alps to the Arctic. Science Advances.

[ref-7] Bertrim C, Aherne J (2023). Moss bags as biomonitors of atmospheric microplastic deposition in urban environments. Biology.

[ref-42] Brahney J, Hallerud M, Heim E, Hahnenberger M, Sukumaran S (2020). Plastic rain in protected areas of the United States. Science (New York, N.Y.).

[ref-9] Cole M, Lindeque P, Halsband C, Galloway TS (2011). Microplastics as contaminants in the marine environment: a review. Marine Pollution Bulletin.

[ref-10] Cowger W, Steinmetz Z, Gray A, Munno K, Lynch J, Hapich H, Primpke S, De Frond H, Rochman C, Herodotou O (2021). Microplastic spectral classification needs an open source community: open specy to the rescue!. Analytical Chemistry.

[ref-53] Dehghani S, Moore F, Akhbarizadeh R (2017). Microplastic pollution in deposited urban dust, Tehran metropolis, Iran. Environmental Science and Pollution Research.

[ref-11] Diana ZT, Chen Y, Rochman CM (2025). Paint: a ubiquitous yet disregarded piece of the microplastics puzzle. Environmental Toxicology and Chemistry.

[ref-54] Dimante-Deimantovica I, Saarni S, Barone M, Buhhalko N, Stivrins N, Suhareva N, Tylmann W, Vianello A, Vollertsen J (2024). Downward migrating microplastics in lake sediments are a tricky indicator for the onset of the Anthropocene. Science Advances.

[ref-12] Dris R, Gasperi J, Saad M, Mirande C, Tassin B (2016). Synthetic fibers in atmospheric fallout: a source of microplastics in the environment?. Marine Pollution Bulletin.

[ref-13] Evangeliou N, Grythe H, Klimont Z, Heyes C, Eckhardt S, Lopez-Aparicio S, Stohl A (2020). Atmospheric transport is a major pathway of microplastics to remote regions. Nature Communications.

[ref-57] Gitlitz J, Gartner E, Vallette J (2024). Vinyl Chloride: The Poison That Makes The Plastic. https://www.beyondplastics.org/publications/vinyl-chloride-poison-that-makes-plastic.

[ref-8] Government of Canada (2023). Canadian climate normals 1991–2020 Data [Data set].

[ref-14] Gunawardana C, Goonetilleke A, Egodawatta P, Dawes L, Kokot S (2012). Source characterisation of road dust based on chemical and mineralogical composition. Chemosphere.

[ref-48] Hammer Ø, Harper DAT, Ryan PD (2001). PAST: paleontological statistics software package for education and data analysis. Palaeontologia Electronica.

[ref-56] Huntington A, Corcoran PL, Jantunen L, Thaysen C, Bernstein S, Stern GA, Rochman CM (2020). A first assessment of microplastics and other anthropogenic particles in Hudson Bay and the surrounding eastern Canadian Arctic waters of Nunavut. FACETS.

[ref-15] Hurley RR, Lusher AL, Olsen M, Nizzetto L (2018). Validation of a method for extracting microplastics from complex, organic-rich, environmental matrices. Environmental Science & Technology.

[ref-16] Kang H, Park S, Lee B, Kim I, Kim S (2022). Concentration of microplastics in road dust as a function of the drying period—a case study in G city, Korea. Sustainability.

[ref-49] Kosuth M, Mason SA, Wattenberg EV (2018). Anthropogenic contamination of tap water, beer, and sea salt. PLOS ONE.

[ref-45] Koutnik VS, Alkidim S, Leonard J, Deprima F, Cao S, Hoek EMV, Mohanty SK (2021). Unaccounted microplastics in wastewater sludge: where do they go? ACS Environmental Science and Technology Water.

[ref-17] Knight LJ, Parker-Jurd FNF, Al-Sid-Cheikh M, Thompson RC (2020). Tyre wear particles: an abundant yet widely unreported microplastic?. Environmental Science and Pollution Research.

[ref-18] Leads RR, Weinstein JE (2019). Occurrence of tire wear particles and other microplastics within the tributaries of the Charleston Harbor Estuary, South Carolina, USA. Marine Pollution Bulletin.

[ref-46] Li C, Gan Y, Zhang C, He H, Fang J, Wang L, Wang Y, Liu J (2021). “Microplastic communities” in different environments: differences, links, and role of diversity index in source analysis. Water Research.

[ref-19] Lim X (2021). Microplastics are everywhere—but are they harmful?. Nature.

[ref-20] MacLeod M, Arp HPH, Tekman MB, Jahnke A (2021). The global threat from plastic pollution. Science.

[ref-21] Monira S, Bhuiyan MA, Haque N, Shah K, Roychand R, Hai FI, Pramanik BK (2021). Understanding the fate and control of road dust-associated microplastics in stormwater. Process Safety and Environmental Protection.

[ref-47] Mutshekwa T, Mulaudzi F, Maiyana VP, Mofu L, Munyai LF, Murungweni FM (2025). Atmospheric deposition of microplastics in urban, rural, forest environments: a case study of Thulamela Local Municipality. PLOS ONE.

[ref-22] O’Brien S, Okoffo ED, Rauert C, O’Brien JW, Ribeiro F, Burrows SD, Toapanta T, Wang X, Thomas KV (2021). Quantification of selected microplastics in Australian urban road dust—ScienceDirect. Retrieved May 9, 2022. https://www.sciencedirect.com/science/article/pii/S0304389421007755?casa_token=h48_J_wMWnUAAAAA:ly4cmi1bsDPl3mXp4czbGI5kh8TGkeja_iEQ1q0qTaZV0HUX6WodAcCLnGdbltA1naJqAipzDw.

[ref-37] Özen HA, Mutuk T (2025). The influence of road vehicle tyre wear on microplastics in a high-traffic university for sustainable transportation. Environmental Pollution.

[ref-23] Patchaiyappan A, Dowarah K, Zaki Ahmed S, Prabakaran M, Jayakumar S, Thirunavukkarasu C, Devipriya SP (2021). Prevalence and characteristics of microplastics present in the street dust collected from Chennai metropolitan city, India. Chemosphere.

[ref-41] Rezaei M, Riksen MJPM, Sirjani E, Sameni A, Geissen V (2019). Wind erosion as a driver for transport of light density microplastics. Science of the Total Environment.

[ref-24] Roblin B, Ryan M, Vreugdenhil A, Aherne J (2020). Ambient atmospheric deposition of anthropogenic microfibers and microplastics on the Western Periphery of Europe (Ireland). Environmental Science & Technology.

[ref-25] Rochman CM, Brookson C, Bikker J, Djuric N, Earn A, Bucci K, Athey S, Huntington A, McIlwraith H, Munno K, De Frond H, Kolomijeca A, Erdle L, Grbic J, Bayoumi M, Borrelle SB, Wu T, Santoro S, Werbowski LM, Zhu X, Giles RK, Hamilton BM, Thaysen C, Kaura A, Klasios N, Ead L, Kim J, Sherlock C, Ho A, Hung C (2019). Rethinking microplastics as a diverse contaminant suite. Environmental Toxicology and Chemistry.

[ref-26] Rødland ES, Lind OC, Reid MJ, Heier LS, Okoffo ED, Rauert C, Thomas KV, Meland S (2022). Occurrence of tire and road wear particles in urban and peri-urban snowbanks, and their potential environmental implications. Science of the Total Environment.

[ref-27] Simon M, van Alst N, Vollertsen J (2018). Quantification of microplastic mass and removal rates at wastewater treatment plants applying Focal Plane Array (FPA)-based Fourier Transform Infrared (FT-IR) imaging. Water Research.

[ref-28] Teuten EL, Saquing JM, Knappe DRU, Barlaz MA, Jonsson S, Björn A, Rowland SJ, Thompson RC, Galloway TS, Yamashita R, Ochi D, Watanuki Y, Moore C, Viet PH, Tana TS, Prudente M, Boonyatumanond R, Zakaria MP, Akkhavong K, Ogata Y, Hirai H, Iwasa S, Mizukawa K, Hagino Y, Imamura A, Saha M, Takada H (2009). Transport and release of chemicals from plastics to the environment and to wildlife. Philosophical Transactions of the Royal Society B: Biological Sciences.

[ref-55] Thornton Hampton LM, Lowman H, Coffin S, Darin E, De Frond H, Hermabessiere L, Miller E, de Ruijter VN, Faltynkova A, Kotar S, Monclús L, Siddiqui S, Völker J, Brander S, Koelmans AA, Rochman CM, Wagner M, Mehinto AC (2022). A living tool for the continued exploration of microplastic toxicity. Microplastic and Nanoplastic.

[ref-29] Tian M, Morais CLM, Shen H, Pang W, Xu L, Huang Q, Martin FL (2022). Direct identification and visualisation of real-world contaminating microplastics using Raman spectral mapping with multivariate curve resolution-alternating least squares. Journal of Hazardous Materials.

[ref-30] Walvoort D, Brus D, Gruijter JD (2023). spcosa: spatial coverage sampling and random sampling from compact geographical strata (Version 0.4-2) [Computer software]. https://cran.r-project.org/web/packages/spcosa/index.html.

[ref-31] Wang T, Niu S, Wu J, Yu J (2022). Seasonal and daily occurrence of microplastic pollution in urban road dust. Journal of Cleaner Production.

[ref-32] Welsh B, Aherne J, Paterson AM, Yao H, McConnell C (2022). Atmospheric deposition of anthropogenic particles and microplastics in south-central Ontario, Canada. The Science of the Total Environment.

[ref-33] Wilkinson T, Järlskog I, de Lima JA, Gustafsson M, Mattsson K, Andersson Sköld Y, Hassellöv M (2023). Shades of grey—tire characteristics and road surface influence tire and road wear particle (TRWP) abundance and physicochemical properties. Frontiers in Environmental Science.

[ref-43] Wright SL, Ulke J, Font A, Chan KLA, Kelly FJ (2020). Atmospheric microplastic deposition in an urban environment and an evaluation of transport. Environment International.

[ref-34] Xu Y, Rillig MC, Waldman WR (2022). New separation protocol reveals spray painting as a neglected source of microplastics in soils. Environmental Chemistry Letters.

[ref-35] Yukioka S, Tanaka S, Nabetani Y, Suzuki Y, Ushijima T, Fujii S, Takada H, Van Tran Q, Singh S (2020). Occurrence and characteristics of microplastics in surface road dust in Kusatsu (Japan), Da Nang (Vietnam), and Kathmandu (Nepal). Environmental Pollution.

[ref-36] Zhang Z, Li P, Hu W, Li J, Li H, Wang R, Li Q, Zou X, Zhou B, Chang C, Guo Z (2025). Electric forces can enhance the emission of microplastics into air. Environmental Pollution.

