# Supplementary figures and images for: Are northern communities an overlooked source of microplastics and tire wear particles in the Arctic?
Source: PeerJ. 2025 Oct 23;13:e20237. doi: 10.7717/peerj.20237 (PMC12554306; doi:10.7717/peerj.20237)

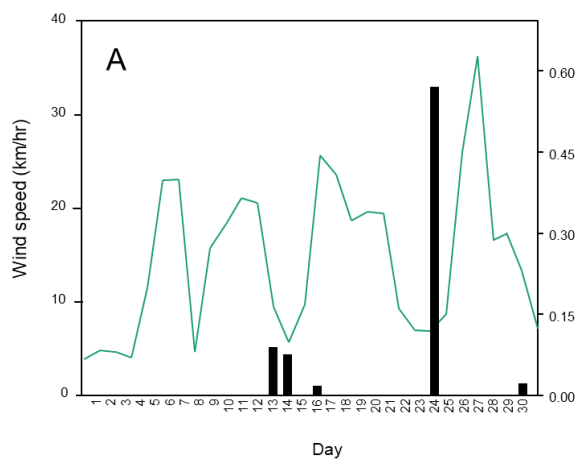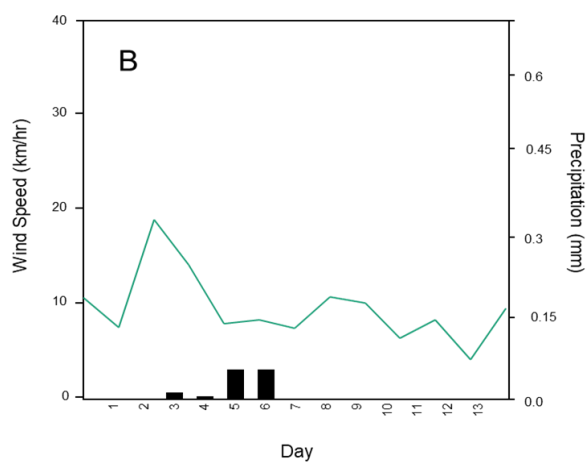

C

Supplement: Supplemental Information 1 — Wind speed (km/hr, blue line) and precipitation (mm, black bars) for A) June 2022 and B) July 2022 prior to sampling period in Iqaluit, Nunavut. Data obtained from Iqaluit Climate Air Monitoring Station. [file peerj-13-20237-s001.pdf]
